# Sialylated immunoglobulin G can neutralize influenza virus infection through receptor mimicry

**DOI:** 10.18632/oncotarget.7244

**Published:** 2016-02-08

**Authors:** Tao Huang, Xueling Chen, Conghui Zhao, Xingmu Liu, Zaiping Zhang, Tongfei Li, Ruiman Sun, Huan Gu, Jiang Gu

**Affiliations:** ^1^ Department of Pathology and Provincial Key Laboratory of Infectious Diseases and Immunopathology, Collaborative and Creative Center, Shantou University Medical College, Shantou, Guangdong, 515041, China; ^2^ Department of Oral Pathology, Beijing Stomatological Hospital, Capital Medical University, Beijing, 100050, China; ^3^ Department of Pathology, Beijing University Health Science Center, Beijing, 100083, China; ^4^ Department of General Surgery, Second Affiliated Hospital, Shantou University Medical College, Shantou, Guangdong, 515041, China

**Keywords:** IVIG, sialylation, influenza virus, neutralizing activity, receptor

## Abstract

Influenza viruses possess a great threat to human health, but there is still no effective drug to deal with the outbreak of possible new influenza subtypes. In this study, we first fractionated sialylated immunoglobulin G (IgG), mainly Fab sialylated fraction, with sambucus nigra agglutinin affinity chromatography. We then demonstrated that sialylated IgG possessed more effective neutralizing activity against 2009 A (H1N1) subtype than that of IgG mixture, and sialosides on the Fab is crucial in this neutralization reaction as when such residues were removed with neuraminidase A digestion the blocking effect was significantly reduced. It appears that sialic acid residues attached to Fab could serve as binding moieties to receptor binding site of influenza virus. These findings indicate that sialylated IgG probably is an effective anti-influenza broad-spectrum drug utilizing its receptor mimicry to competitively inhibit the attachment of influenza viruses with sialic acid receptors on target cells. This property would be particularly useful if it can be applied to prevent newly emerged influenza virus strain infections in future epidemics.

## INTRODUCTION

Influenza viruses are enveloped negative-stranded RNA viruses possessing a great threat to human health. There have been four influenza pandemics since 1918 including these occurred in 1918, 1957, 1968 and 2009 [1]. The reported human cases infected with new avian-origin influenza subtypes including 1999 H9N2, 2005 H5N1, 2013 H7N9, 2013 H6N1 and 2014 H10N8 have been on the increase in the last two decades [2]. This increase further highlights the urgency and importance of prevention and treatment of possible next pandemics by new variants of influenza viruses.

It was suggested that the precondition for an avian influenza virus infection and transmission in humans is the alteration of its receptor preference from α2-3- linked sialosides (avian influenza receptors) to α2-6- linked sialosides (human influenza receptors) [3], and the attachment coordinated by viral surface hemagglutinin (HA) and cell surface receptors is the essential first step for influenza virus infection of target cells [4], Therefore, utilization of soluble sialic acid-containing macromolecules to competitively combine with viral HA could be a hopeful strategy for prevention and treatment of influenza viral infection.

As the key player of humoral immune response, it has long been known that IgG molecules are glycoproteins [5]. The asparagine 297 (Asn 297) in the C_H_2 domains of the Fc region is the conservative glycosylation site, extra N-glycans possibly attach to the variable regions of the IgG Fab portions, and about 15% to 25% normal human IgG Fab bear N-linked oligosaccharides [6-8]. Human IgG-Fc oligosaccharide is of the biantennary complex type with a core heptasaccharide and variable addition of outer arm sugar residues [9]. The glycans of the Fab are of biantennary complex type too, with highly sialylated residues in contrast to Fc glycans [10, 11]. If IgG Fab sialosides could react with HA, sialylated IgG will probably be an effective and broad-spectrum anti-influenza molecule in light of its subsequent powerful clearance mechanisms activated by Fc regions including antibody dependent cellular cytotoxicity (ADCC), complement dependent cellular cytotoxicity (CDC) and phagocytosis etc [5, 12,13].

In this study, sialylated IgG was first fractionated with sambucus nigra agglutinin (SNA) affinity chromatography from purchased intravenous immunoglobulin G (IVIG) (Shanghai RAAS, China). In consistent with the reports of Johannes Stadlmann etc [14], the binding fractions of IVIG including elution fraction 1 (E1) IVIG and elution fraction 2 (E2) IVIG with SNA agarose column were mainly bound by Fab sialylation. The more effective neutralizing activity against 2009 A (H1N1) subtype of sialylated IgG including E1 IVIG and E2 IVIG in comparison to IVIG mixture and flow through fraction (FT) IVIG was demonstrated with real time PCR and Western blot after infection of A549 or Madin-Darby canine kidney cells (MDCK cells). In addition, the reaction of influenza virus with sialylated IgG through sialic acid residues on IgG molecules was further established by reduced neutralizing activity after desialylation of sialylated IVIG with neuraminidase (NA) digestion. These results indicate that sialylated IVIG probably is an effective anti-influenza broad-spectrum drug utilizing its receptor mimicry to competitively inhibit the attachment of influenza viruses to sialic acid receptors on target cells.

## RESULTS

### Fractionation of IVIG with SNA affinity chromatography

**Figure 1:**
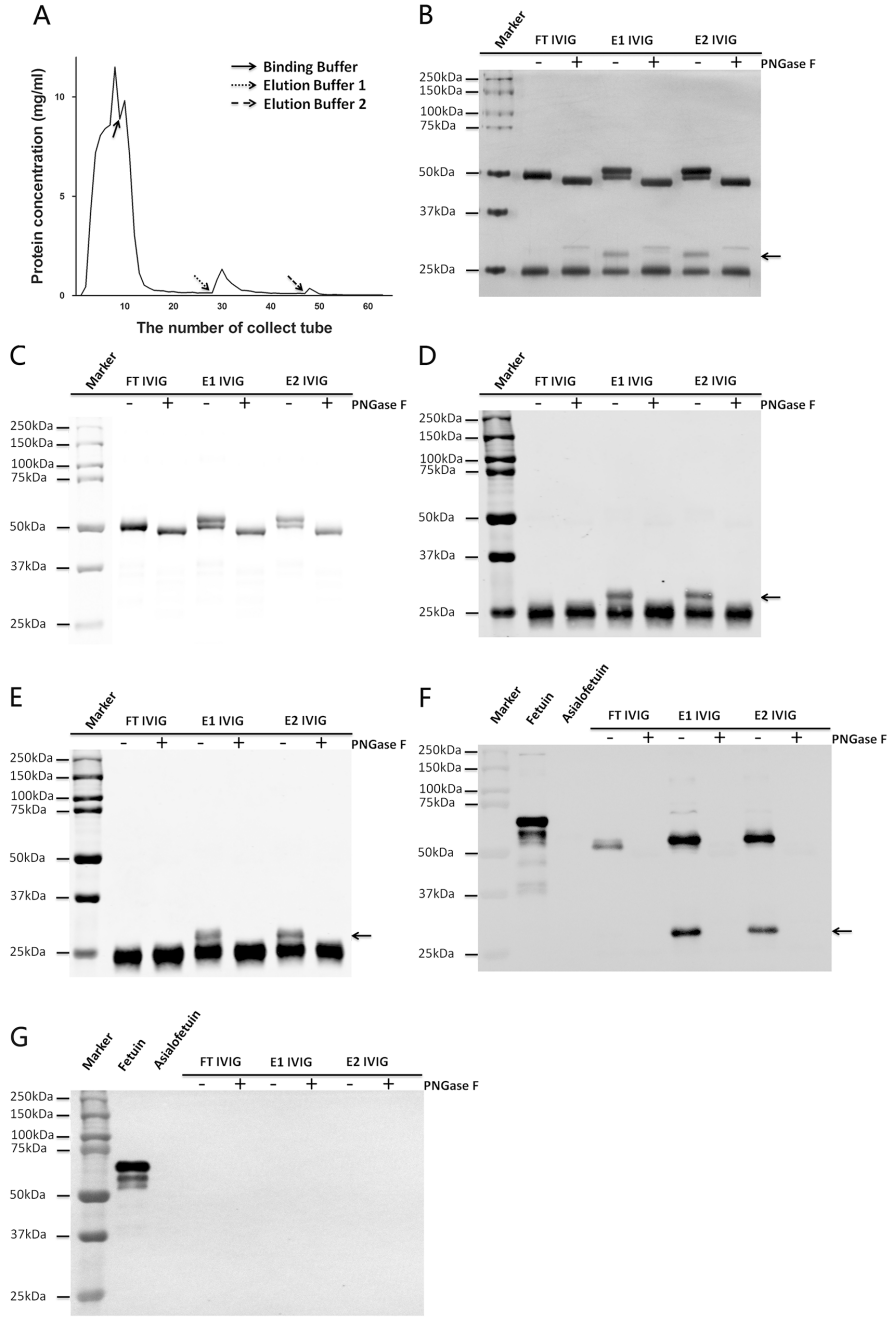
IVIG was fractionated with SNA affinity chromatography **A.** Typical fractionation chromatogram of IVIG on a SNA agarose column. Three fractions including one SNA non-binding fraction named FT IVIG (elution with Binding Buffer) and two SNA binding fractions separately named E1 IVIG (elution with Elution Buffer 1) and E2 IVIG (elution with Elution Buffer 2) were obtained. **B.** IVIG fractions were separated with SDS-PAGE and stained with silver. Two higher molecular weight bands existed above the 50 kDa heavy chain and the 25 kDa light chain (arrow) respectively of E1 IVIG and E2 IVIG which fell back to the normal molecular weight following deglycosylation with PNGase F. **C–E.** The identities of these two extra bands were established with Western blot. The band above the 50 kDa heavy chain was Ig γ heavy chain (C), the band above the 25 kDa light chain was the mixture of kappa light chain (D) and lambda light chain (E). **F&G.** The presence and the linkage patterns of sialic acids on IVIG fractions were examined with Lectin blot. Sialic acids on IVIG fractions were linked through α-2,6 but not α-2,3, which were conformed with SNA (F) and MAA (G). Strong SNA positive signals existed on both the heavy chain and the light chain of E1 IVIG and E2 IVIG. Weak SNA positive signal existed on the heavy chain of FT IVIG (F). No MAA positive signal was detectable on any IVIG fractions (G). Fetuin and Asialofetuin were used as control glycoproteins.

Lectin affinity chromatography with sialic acid-specific SNA has been extensively applied in enrichment of sialylated IVIG. In earlier studies, the two SNA+ IVIG fractions including E1 IVIG (elution with 0.5 M neutral lactose in pH 7.5 TBS) and E2 IVIG (elution with 0.5 M lactose in 0.2 M acetic acid) were pooled together [14–16]. It is worthy to note that E2 IVIG exerted a more effective anti-inflammatory effect compared to E1 IVIG when E1 and E2 were collected and analyzed separately [17]. In this study, we also collected and analyzed E1 fraction and E2 fraction separately (Figure 1A).

Unlike other immunoglobulin subtypes including IgM, IgA, IgD and IgE, human IgG does not contain O-linked glycans [18]. PNGase F is an amidase that cleaves almost all N-link carbohydrates between the innermost GlcNAc and Asn residues from glycoproteins [19]. It would be an effective method to determine the contribution of carbohydrates in the molecular weight by comparing the difference of before and after PNGase F digested fractions. The difference in molecular weight of the three IVIG fractions (FT, E1 and E2 IVIG) were first assessed with reducing SDS-PAGE followed by silver stain, Western blot or Lectin blot. With silver stain analysis (Figure 1B), higher molecular weight bands above the 50 kDa heavy chain and the 25 kDa light chain of E1 IVIG and E2 IVIG fractions must be due to extra N-link carbohydrates attachment, which fell back to the normal molecular weight following deglycosylation with PNGase F. With Western blot analysis, these extra bands were confirmed to be IgG heavy chains (above the 50 kDa band) and Igκ or λ light chain (above the 25 kDa band) respectively (Figure 1C–1E). The carbohydrates attached to these bands were terminal sialylation linked with α-2,6 but not α-2,3 as shown by Lectin blot with biotinylated SNA and MAA (Figure 1F&1G). In addition, the denatured and reduced heavy chain of FT IVIG also reacted with SNA although weaker than E1 and E2 IVIG indicating that FT IVIG was not non-sialylated IVIG. The oligosaccharides attached at Asn 297 are “enclosed” within the protein structure to define protein spatial structure [20]. So these sialylated glycans of FT IVIG appear to be inaccessible by SNA in natural state, but are exposed under denaturation and reduction state and accessible by SNA.

### SNA affinity chromatography mainly enriched Fab sialylated IVIG

IVIG fractions were further enzyme-digested into Fab and Fc (contain undigested IVIG) fragments using papain to locate the site of sialic acid contributing to the binding with SNA agarose column of E1 and E2 IVIG.

**Figure 2:**
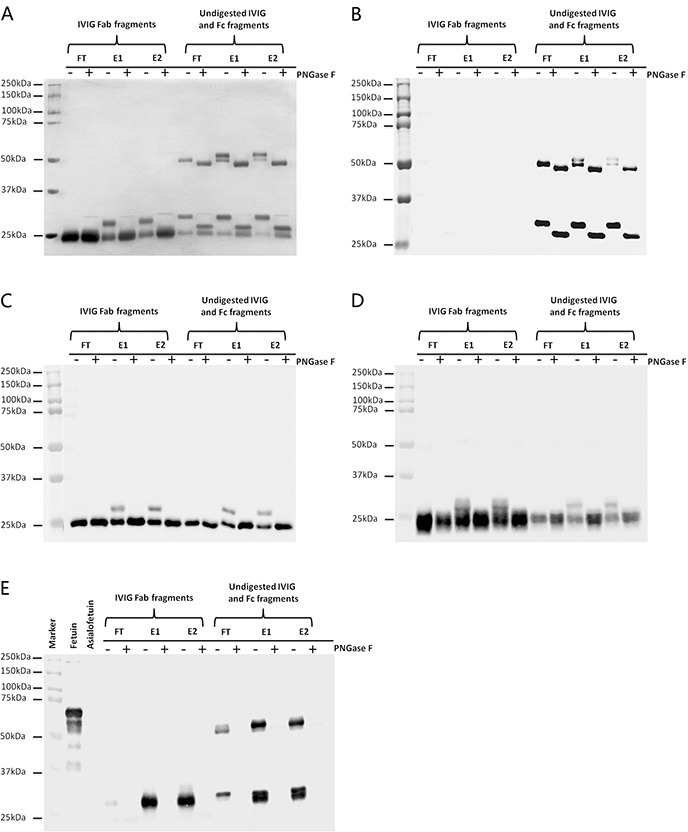
SNA affinity chromatography mainly enriched Fab sialylated IVIG FT IVIG, E1 IVIG and E2 IVIG were digested with papain to produce Fab and Fc (contain undigested IVIG) fragments. Silver stain **A.** Western blot with anti-IgG γ heavy chain antibody **B.** anti-Ig κ light chain antibody **C.** and anti-Ig λ light chain antibody **D.** and Lectin blot with SNA **E.** were performed to analysis the affinity characteristic of SNA agarose column with IVIG. The higher molecular weight bands above the Fab fragments of E1 IVIG and E2 IVIG (A) were the mixture of IgG γ heavy chain Fd fragment but not Fc fragment (B), kappa light chain (C) and lambda light chain (D) with extra N-linked oligosaccharides. These bands with higher molecular weight fell back to the normal molecular weight following deglycosylation with PNGase F. (E) All sialylated Fab fragments were enriched to E1 IVIG or E2 IVIG fraction, but Fc fragments of FT IVIG, E1 IVIG and E2 IVIG all possessed sialylated portion, which were conformed with SNA blot. Fetuin and Asialofetuin were used as positive and negative controls of α-2,6 linked sialylated glycoproteins.

With silver stain analysis (Figure 2A), additional higher molecular weight bands in E1 and E2 IVIG Fab fragments were generated comparing with FT IVIG Fab fragments and these higher molecular weight bands fell back to the normal molecular weight following enzyme digestion with PNGase F. In contrast, there was no molecular weight difference among FT, E1 and E2 IVIG Fc fragments. As shown in Figure 2B–2D, these additional higher molecular weight bands in E1 and E2 IVIG Fab fragments were the mixture of IVIG heavy chain Fd fraction not Fc fraction (Figure 2B), κ light chain (Figure 2C) and λ light chain (Figure 2D). No SNA reactive Fab fragments, additional higher molecular weight bands, were detected in FT IVIG Fab fragments indicating that all Fab sialylated IVIG were enriched by SNA affinity chromatography (Figure 2E). The positive reaction of FT IVIG Fc fragments with SNA under denaturation and reduction state further affirmed the intramolecular location of Asn 297 oligosaccharides in natural state (Figure 2E).

Based on these findings, SNA affinity chromatography mainly enriched Fab sialylated IgG. Part sialic acids located at Fc Asn 297 may also bind to SNA agarose column in natural state, but the contribution should be far below the contribution of Fab sialic acids.

### The neutralizing activity of IgG mixture against 2009 A (H1N1) subtype

**Figure 3:**
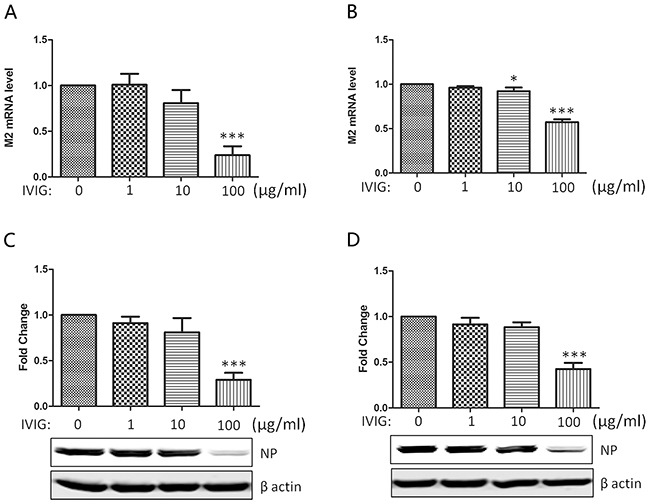
The neutralizing activity of IVIG against 2009 A (H1N1) influenza virus subtype The influenza virus was preincubated with IVIG at indicated doses for 30 minutes before infection of MDCK **A&C.** or A549 cells **B&D.** The expression of viral matrix 2 (M2) mRNA and virus Nucleoprotein (NP) in infected MDCK cells (A&C) and A549 cells (B&D) were examined with real time qPCR and Western blot 24 h post infection. Reduction of M2 and NP expression indicated the neutralizing activity of IVIG. Data was presented as means ± S.D. of four independent experiments. The significant level was set at *, p<0.05; ***, p<0.001.

IVIG is a product pooled and manufactured from more than 1000 healthy donors’ plasma. Because most donors very likely have been infected with seasonal influenza viruses and vaccinated, it would be understandable that IVIG contains antibodies against influenza viruses in spite of sialylated Fab. In fact, the protective role of IVIG against seasonal H1N1 and pandemic H1N1 2009 viruses has been reported previously [21–23]. In this study, virus was preincubated with different doses of IVIG first to determine whether IVIG purchased from Shanghai RAAS, China possesses antibodies against Influenza A/Nanchang/8002/2009 (H1N1) virus strain. As shown in Figure 3, the expressions of viral matrix 2 (M2) mRNA and virus nucleoprotein (NP) in infected MDCK cells (A&C) and A549 cells (B&D) were gradually reduced along with the increase of IVIG indicating the neutralizing activity of IVIG against 2009 A (H1N1) subtype. Remarkable neutralizing activity was identified in the 100 μg/ml dosage group, we adopted this concentration to further compare the neutralizing activities between SNA- IVIG and SNA+ IVIG.

### Neutralizing activity of IgG was enhanced after fractionated with SNA affinity chromatography

**Figure 4:**
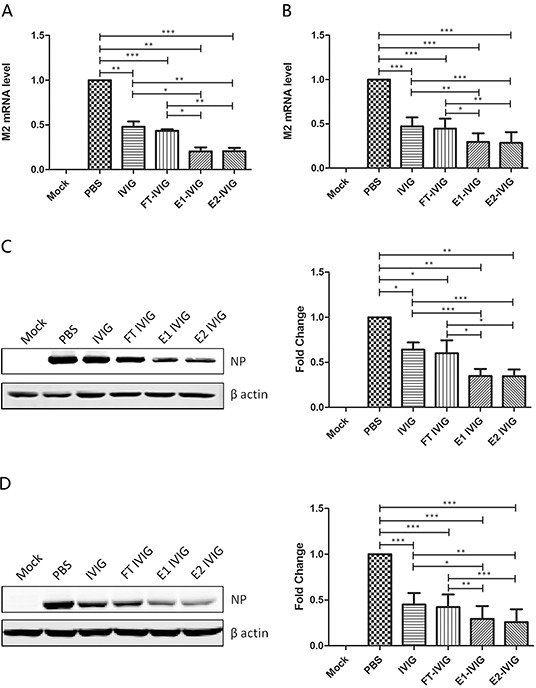
Virus neutralizing activity of SNA+ IVIG is more effective than that of IVIG and SNA− IVIG Virus was preincubated with 100 μg/ml IVIG, FT IVIG, E1 IVIG or E2 IVIG for 30 minutes before infection of MDCK or A549 cells. No virus infection (Mock) and PBS-preincubated virus were used as negative and positive controls respectively. The expressions of viral M2 mRNA and virus NP in infected MDCK cells **A&C.** and A549 cells **B&D.** were examined with real time qPCR and Western blot 24 h post infection. Decreased M2 and NP expression in IVIG and FT IVIG groups in comparison to PBS group indicated the existence of antibodies against influenza virus in IVIG through the Fab variable region. Lower M2 and NP expression levels showed a more effective neutralizing activity of SNA+ IVIG (E1 IVIG and E2 IVIG) in comparison with IVIG and SNA- IVIG (FT IVIG). Data was presented as means ± S.D. of four independent experiments. The significant level was set at *, p<0.05; **, p<0.01; ***, p<0.001.

**Figure 5:**
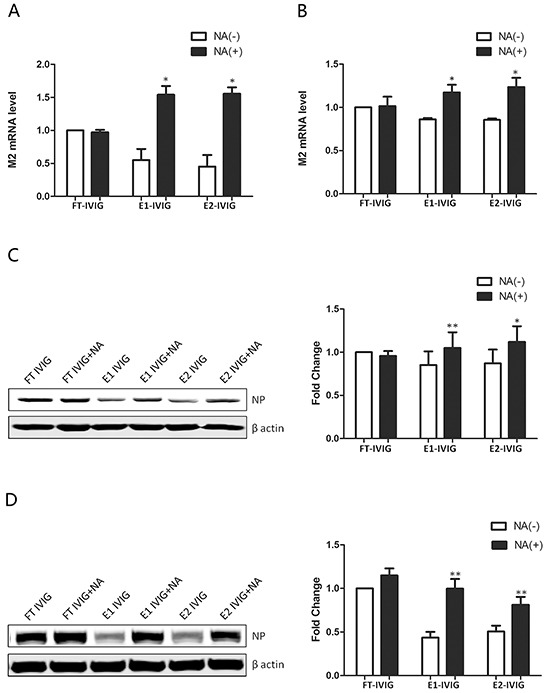
The neutralizing activity of SNA+ IVIG was reduced following desialylation with neuraminidase (NA) FT IVIG, E1 IVIG and E2 IVIG were desialylated with NA before preincubation with virus to analyze the effect of sialic acids in neutralizing activity. Higher expressions of viral M2 mRNA and virus NP in infected MDCK cells **A&C.** and A549 cells **B&D.** following treatment by E1 IVIG NA (+) and E2 IVIG NA (+) in comparison with E1 IVIG NA (−) and E2 IVIG NA (−) indicate the effect of sialic acids in neutralizing activity of SNA+ IVIG. No significant difference in M2 and NP expressions in FT IVIG groups before and after treatments with NA, both of which were lower than that of the PBS group, further proved that the neutralizing effect of this fraction was derived from Fab variable region rather than from the sialic acids attached to the Fab. NA (+): with neuraminidase digestion; NA (−): without neuraminidase digestion. Data was presented as means ± S.D. of four independent experiments. The significant level was set at *, p<0.05 and **, p<0.01.

The comparison of neutralizing activity among IVIG and its fractions including FT IVIG, E1 IVIG and E2 IVIG were performed through examining the changes of viral M2 mRNA and virus NP in infected MDCK cells (A&C) and A549 cells (B&D) with real time qPCR and Western blot (Figure 4). Decreased M2 and NP expression in IVIG and FT IVIG groups in comparison to PBS group indicated the existence of antibodies with their Fab variable regions against influenza viruses in IVIG (Figure 4). There was no significant difference in M2 and NP expression in FT IVIG group before and after treatments with NA, both of which were lower than that of the PBS group. This further proved that the neutralizing effect of this fraction should be derived from Fab variable region rather than from the sialic acids attached to the Fab (Figure 5).

Lower M2 and NP expression levels showed a more effective neutralizing activity of SNA+ IVIG (E1 IVIG and E2 IVIG) in comparison to IVIG and SNA- IVIG (FT IVIG) (Figure 4). In order to determine whether the more effective neutralizing activity of SNA+ IVIG was derived from the reaction between viral HA and sialosides of SNA+ IVIG but not an antibody-antigen reaction, FT IVIG, E1 IVIG and E2 IVIG were desialylated using NA before preincubation with the virus. As shown in Figure 5, the neutralizing activity of SNA+ IVIG (E1 IVIG and E2 IVIG) were reduced after desialylation with NA. In contrast, there was no notable change in neutralizing activity of FT IVIG before and after treatment with NA.

## DISCUSSION

IVIG, as a healthy human plasma derived product, has been widely used clinically in the treatment of many diseases including immunodeficiency, autoimmune diseases and inflammatory disorders etc with little side effect [8,24]. IVIG also possesses cross-reactive specific antibodies against viral antigens and its protective role against influenza viruses and clinical utilization in against complications of infection such as severe pneumonitis and influenza encephalopathy have been reported previously [21–23].

In this study, we observed the neutralizing activities of IVIG against Influenza A/Nanchang/8002/2009 (H1N1) virus strain (Figure 3). As IVIG is a mixture of over 1000 healthy individuals, it would be understandable that IVIG contains antibody population against HA, NA or other viral antigens of the virus with the Fab variable region rather than with the sialic acids attached to the Fab. As shown in Figure 4, SNA- IVIG (FT IVIG) could neutralize 2009 A (H1N1) influenza virus strain. The neutralizing effect of this fraction should be derived from Fab variable region rather than from the sialic acids attached to the Fab as there was no significant difference in neutralization of FT IVIG before and after treatments with NA (Figure 5). Therefore, cross-reactive specific antibodies with their Fab variable region against influenza virus exist in IVIG.

Recent studies have much more focused on searching for monoclonal antibodies cross-reacting to influenza virus [25-29]. Despite of the fact that monoclonal antibodies might be more specific and effective to neutralize existing influenza viral strain, they would be powerless in fighting newly emerged variants of influenza viruses at their first encounter.

It is believed that the first step for a wide variety of influenza viruses to infect target cells is attachment coordinated by viral surface protein HA and cell surface receptors sialic acid residues. Based on what we found in this study, it would be a reasonable strategy to prevent or treat influenza virus infection with sialylated IgG to competitively inhibit the attachment of influenza viruses to target cells. In this study, we first purified sialylated IgG with lectin chromatography, and established that sialosides attached to Fab fragments were the main contributors to the binding with SNA in affinity chromatography. The characteristic of glycans attached to Fab fragments, which are exposed on the surface of the IgG molecules accessible to lectin and other proteins, renders them capable of binding to viral surface protein HA but not to hinder the reaction between Fc fragments and Fc receptors to activate clearance mechanisms.

The oligosaccharides attached at Asn 297 of IgG Fc fragment are “enclosed” within the protein structure [20]. The positive reaction of FT IVIG Fc fragments with SNA under denaturation and reduction state (Figure 2E) further affirmed the intramolecular location of Asn 297 oligosaccharides in natural state. Thereby, SNA affinity chromatography mainly enriched Fab sialylated IgG. Portions of sialic acids located at Fc Asn 297 may also bind to SNA agarose column in natural state, but the contribution should be far below that of Fab sialic acids.

The receptor binding site (RBS) of influenza viruses is a shallow pocket located on the surface of HA, and the interactions between the amino acid residues of the RBS and the glycan ligands of receptors are weak (K_diss_ > 0.1 mM) [30]. The specificity and affinity of HA for its glycan ligand are related not only to the type of sialic acid linkage (α2-3 or α2-6) but also to glycan modifications including fucosylation, sulphation and additional sialylation [31-33]. It would be an important strategy to use gene recombination technology to increase the number of glycans and alter the configuration of sialyloligosaccharide moieties located on the Fab fragments to enhance the affinity and avidity of IgG in its reaction to HA.

In conclusion, we found that sialylated IgG could neutralize 2009 A (H1N1) influenza virus via its Fab attached sialosides binding to viral surface protein HA. This would provide a hopeful strategy to treat or prevent influenza viral infection with sialylated IgG in view of the fact that IVIG have been used clinical to treat a variety of diseases with little side effect for over 35 years. This would be particularly useful if it can be successfully utilized to prevent infection by newly emerged influenza virus strains in future epidemics which pose a great threat to human health.

## MATERIALS AND METHODS

### Ethics statement

All experiments in this study were approved by the Ethics Committee of Shantou University Medical College, China.

### SNA affinity chromatography

Experiment required normal human IVIG was purchased from Shanghai RAAS, China. Before fraction, the pH of solution should be adjusted to 7.0 with 1 M Tris Base (Sangon Biotech company, Shanghai, China). Fraction was performed by lectin affinity chromatography with sialic acid specific SNA following the manufactuer's description (Vector labs, California, USA). In brief, IVIG was diluted with Binding Buffer (20 mM Tris-Hcl pH7.5 containing 0.5 M NaCl and 0.1 M CaCl_2_) and loaded onto SNA agarose column. The column was then washed with 5 bed volumes Binding Buffer to remove the rest of unbound IgG, all these unbound IgG named as flow through IVIG (FT IVIG). The bound IVIG (SNA+ IVIG) was further orderly eluted with Elution Buffer 1 (0.5 M lactose pH7.5 containing 20 mM Tris-Hcl and 0.2 M NaCl) and Elution Buffer 2 (0.5 M lactose in 0.2 M acetic acid). These two SNA+ IVIG were separatelynamed Elution 1 IVIG (E1 IVIG) and Elution 2 IVIG (E2 IVIG). The pH of E2 IVIG need to be adjusted to 7.0 with 1 M Tris Base after collected. The three fractions were concentrated with Amicon Ultra-4 Centrifugal Filter Units, 10kDa (Millipore, Massachusetts, USA) and desalted with Zeba^TM^ Desalt Spin Columns (Pierce Biotechnology, Illinois, USA). The protein concentration was measured by absorbance at 280 nm with Thermo Scientific NanoDrop 2000/2000C (Thermo Scientific, Illinois, USA).

### Production of igG Fab and Fc fragments

Fab and Fc fragments were produced from normal human IVIG by papain digestion with Pierce Fab Preparation kit following the manufacturer's instructions (Thermo Scientific, Illinois, USA).

### Enzymatic deglycosylation or desialylation

IVIG including FT, E1 and E2 as well as their Fab and Fc fragments were deglycosylated with PNGase F (NEB, Massachusetts, USA) according to manufacturer's instructions. Different fractions of IVIG were desialylated by enzymatic digestion with α2-3,6,8,9 Neuraminidase A (NA) (NEB, Massachusetts, USA), under the condition of 100μg FT, E1 or E2 IVIG was incubated with 5μl enzyme for 5 h at 37°C.

### Sodium dodecyl sulfate polyacrylamide gel electrophoresis (SDS-PAGE)

The fractions of IVIG and their fragments with or without PNGase F treatment were separated with 10% SDS-PAGE under reducing conditions. In brief, samples were denatured with heating at 100°C for 10 minutes in denaturing buffer (62.5 mM, pH 6.8 Tris-HCl solution containing 10% Glycerol, 2% SDS, and 10 mM DTT), and then loaded into 10% SDS-PAGE gel. The samples were first ran at 80 voltage for 30 minutes, followed by ran at 120 voltage for about 60min till the dye run to the bottom of the gel. After electrophoresis, the gels were stained with silver or transferred to nitrocellulose filter membranes (Whatman, Dassel, Germany) and examined with Western blot or Lectin blot were described below.

### Silver stain

The gels were fixed with fixation fluid (10% acetic acid and 40% ethanol) for 30 minutes, incubated in 30% ethanol solution containing 0.5 M sodium acetate anhydrous and 0.02 M sodium thiosulfate for 30 minutes, washed in deionized water three times for 10 minutes each, stained in 0.01 M silver nitrate solution combining with 0.05% formaldehyde for 40 minutes, visualized with the solution containing 0.24 M sodium carbonate anhydrous and 0.05% formaldehyde and terminated with 5% acetic acid.

### Western blot

For IVIG fractions and their fragments, loaded 1 μg protein aliquots and Precision Plus protein standards (BIO-RAD, California, USA) onto 10% reducing SDS-PAGE gels. After electrophoresis, the separated proteins were transferred to nitrocellulose filter membranes. Rabbit anti-human IgG γ Fc region antibody (Dako, Copenhagen, Denmark), mouse anti-human Ig κ chainantibody (ZSGB-BIO, Beijing, China) and mouse anti-human Ig λ chain antibody (ZSGB-BIO, Beijing, China) at a dilution of 1:1,000 were used as primary antibodies.

For cell lysates of infected A549 or MDCK cells, they were prepared using RIPA buffer (Millipore, Massachusetts, USA), and the concentrations were determined with BCA protein assay kit (Thermo Scientific, Illinois, USA). About 20μg protein aliquots were separated and transferred to nitrocellulose filter membranes. Rabbit anti-Influenza A Virus Nucleoprotein antibody (GeneTex, California, USA) and mouse anti-β actin monoclonal antibody (ZSGB-BIO, Beijing, China) at a dilution of 1:1,000 were used as primary antibodies.

After incubation with appropriate secondary antibodies including Goat anti-rabbit IgG-680 (1:10,000; LI-COR, Nebraska, USA) and goat anti-mouse IgG-680 (1:10,000; LI-COR, Nebraska, USA), the blots were examined using a Odyssey imaging system (LI-COR, Nebraska, USA).

### Lectin blot

Sialylation of IVIG fractions and their fragments were detected with Dig Glycan Differentiation Kit following the manufacturer's instructions (Roche, Basel, Switzerland). Briefly, 1 μg protein aliquots and Precision Plus protein standards (BIO-RAD, California, USA) were loaded onto 10% reducing SDS-PAGE gels. After separation and transfer, the membranes were incubated with SNA and Maackia amurensis agglutinin (MAA) to detect terminal α2-6- linked and α2-3- linked sialosides, respectively. Fetuinwas used as positive control glycoproteins and Asialofetuin was used as negative control glycoproteins.

### Cell culture

The Madin-Darby canine kidney cell line MDCK and human lung adenocarcinoma cell line A549 were obtained from the American Type Culture Collection (ATCC). MDCK was cultured in Dulbecco's Modified Eagle's Medium (DMEM, Invitrogen, California, USA) with 10% FBS (Hyclone, Waltham, MA). A549 was cultured in Dulbecco's Modified Eagle Medium: Nutrient Mixture F-12 medium (DMEM/F-12, Invitrogen, California, USA) with 10% FBS at 37°C in a humidified atmosphere with 5% CO_2_.

### The preparation and infection of influenza virus

The propagation of Influenza A/Nanchang/8002/2009 (H1N1) virus was performed using embryonated eggs, and titers were determined by 50% tissue culture infection dose (TCID50) assay with MDCK cells.

Before infection, virus was thawed in cool water and keep at 4°C. The preincubation of virus with IVIG fractions was performed at 4°C for 30 minutes in the ratio of 4 × 10^4^ virus: 100 μg IVIG in 1 ml viral growth medium. Infection was performed in 12-well tissue culture plate at a multiplicity of infection (MOI) of 0.1 for 2 h at 37°C in the humidified atmosphere with 5% CO_2_. After viral adsorption, unadsorbed viruses were washed away with PBS and the cells were cultured for 24 h, followed by examining with real time PCR and Western blot. No virus infection (Mock) and PBS-preincubated virus were used as negative and positive controls respectively.

### Real-time PCR

At 24 hours post infection, total cellular RNA was extracted with RNAiso Plus kit (Takara, Dalian, China), and concentrations were measured by absorbance at 260 nm using Thermo Scientific NanoDrop 2000/2000C. Reverse transcription was performed using ReverTra Ace qPCR RT Kit (TOYOBO, Osaka, Japan) according to manufacturer's instructions. Real time quantitative PCR analysis was performed with SYBR Premix Ex Taq (Takara, Dalian, China) using Applied Biosystems 7500 Real-Time PCR System (Applied Biosystems, California, USA). The primers used in this study were: Influenza A virus matrix 2 (M2): sense, 5′-GAGGTCGAAACGCCT-3′, antisense, 5′-CTGTTCCTTTCGATATTCTTCCC-3′ [34]; human β-actin: sense, 5′-AGCGAGCATCCCCCAAA GTT-3′, antisense, 5′-GGGCACGAAGGCTCATCATT-3′ [35]; rodent β-actin: sense, 5′-CCAACCGTGAA AAGATGACC-3′, antisense, 5′-ccagaggcatacagggacag-3′ [36]. The relative expression of M2 mRNA was determined with the method of 2^−ΔΔCt^ [37].

### Statistical analysis

Prism software (Graph Pad, California, USA) was used for statistical analysis. All experiments were repeated at least three times. The datas were expressed as the mean ± S.D. and compared with One-way ANOVA. Comparisons of each group were performed with q-test (Newman-Keul's test). The significant level was set at *, p<0.05; **, p<0.01; ***, p<0.001.

## Abbreviations

IVIG: intravenous immunoglobulin G
HA: hemagglutinin
Ig: immunoglobulin
Asn: asparagine
SNA: sambucus nigra agglutinin
MAA: Maackia amurensis agglutinin
FT: flow through fraction
E1: elution fraction 1
E2: elution fraction 2
NA: neuraminidase
M2: matrix 2
RBS: receptor binding site
NP: nucleoprotein
